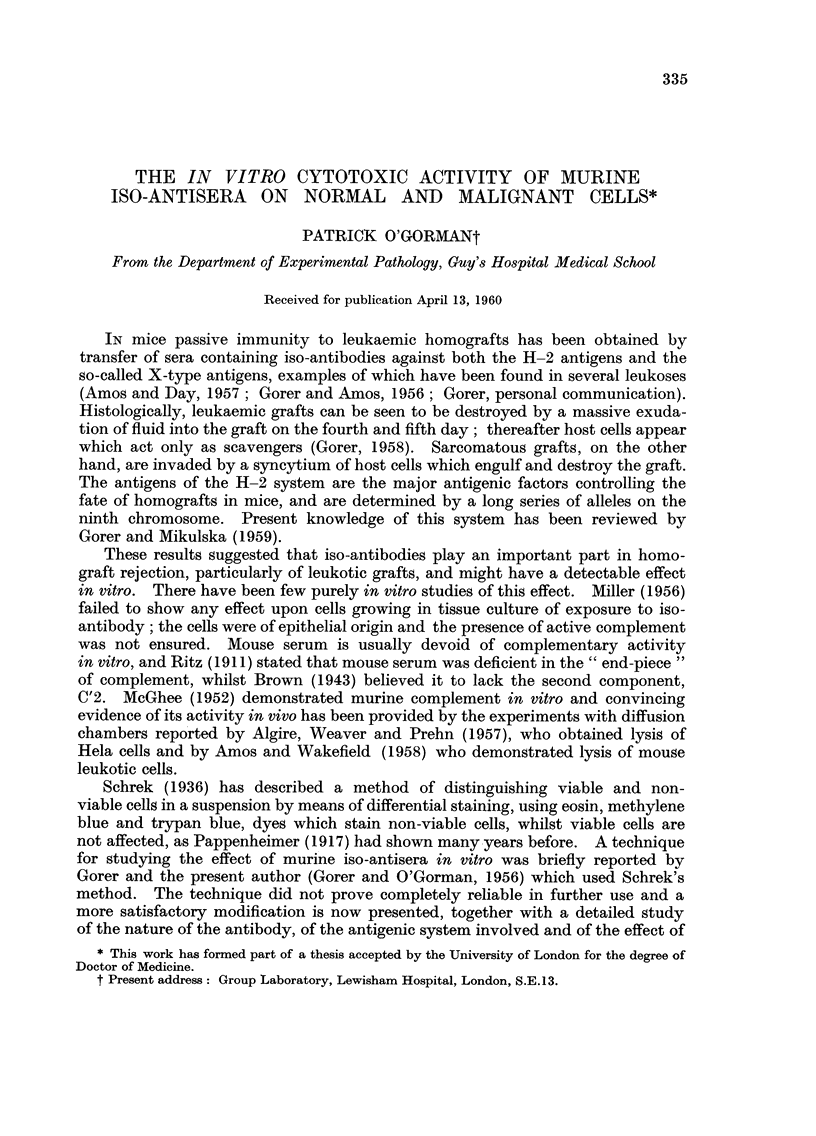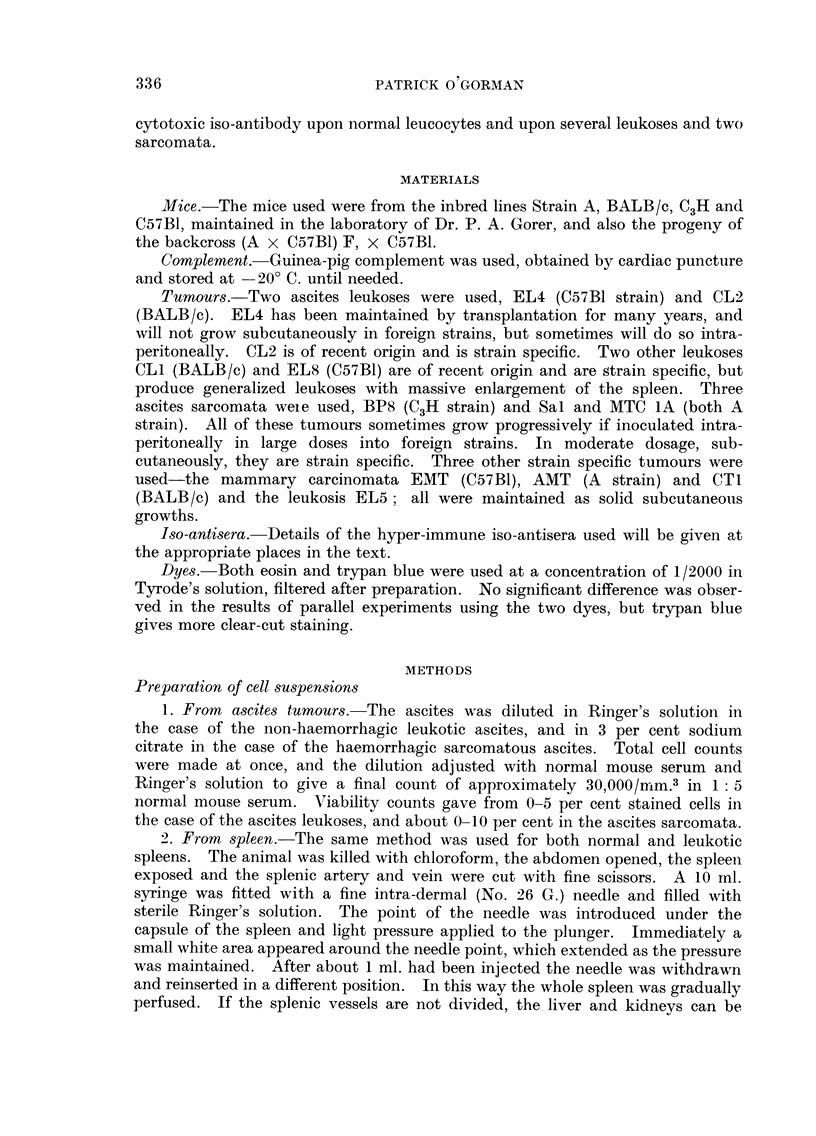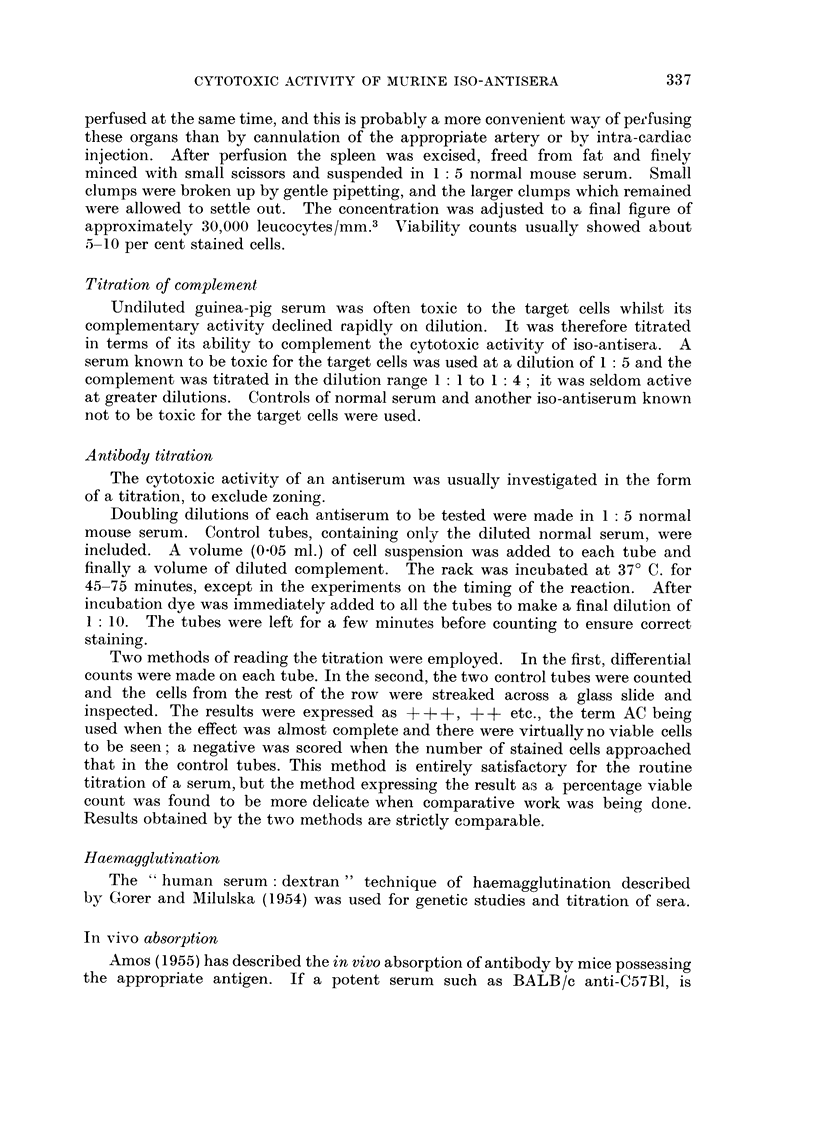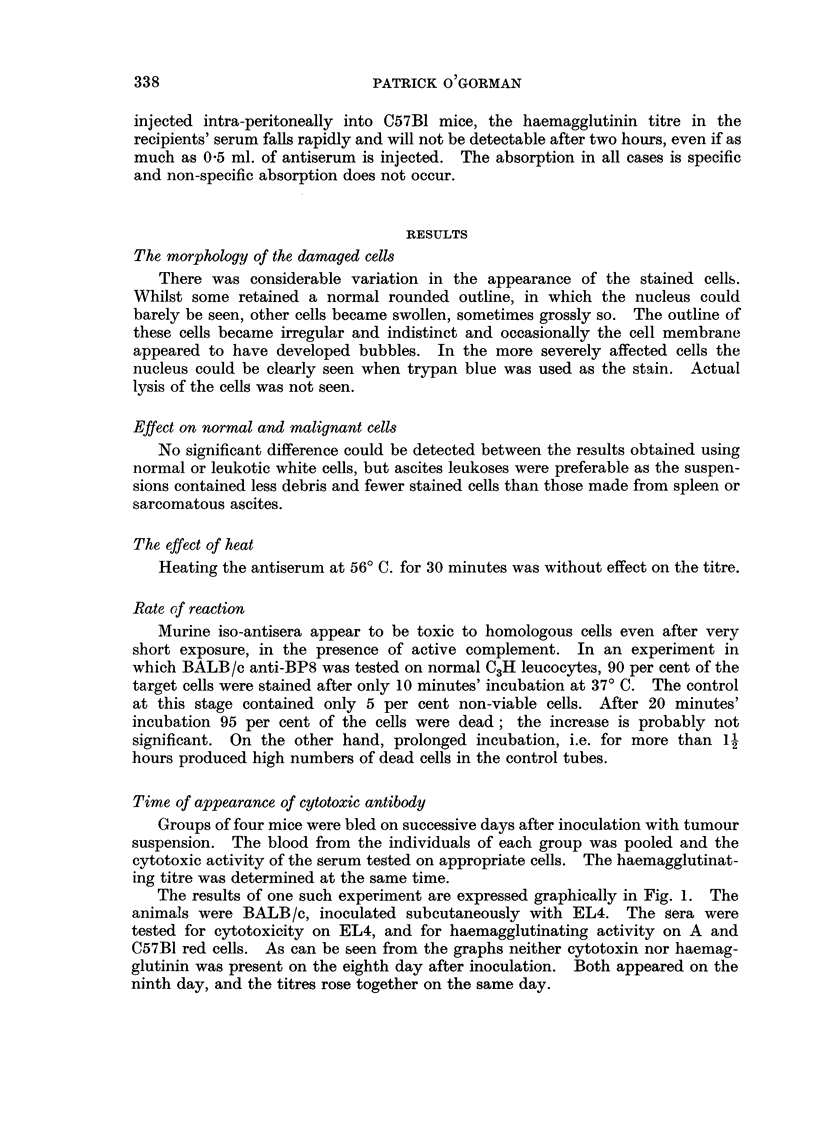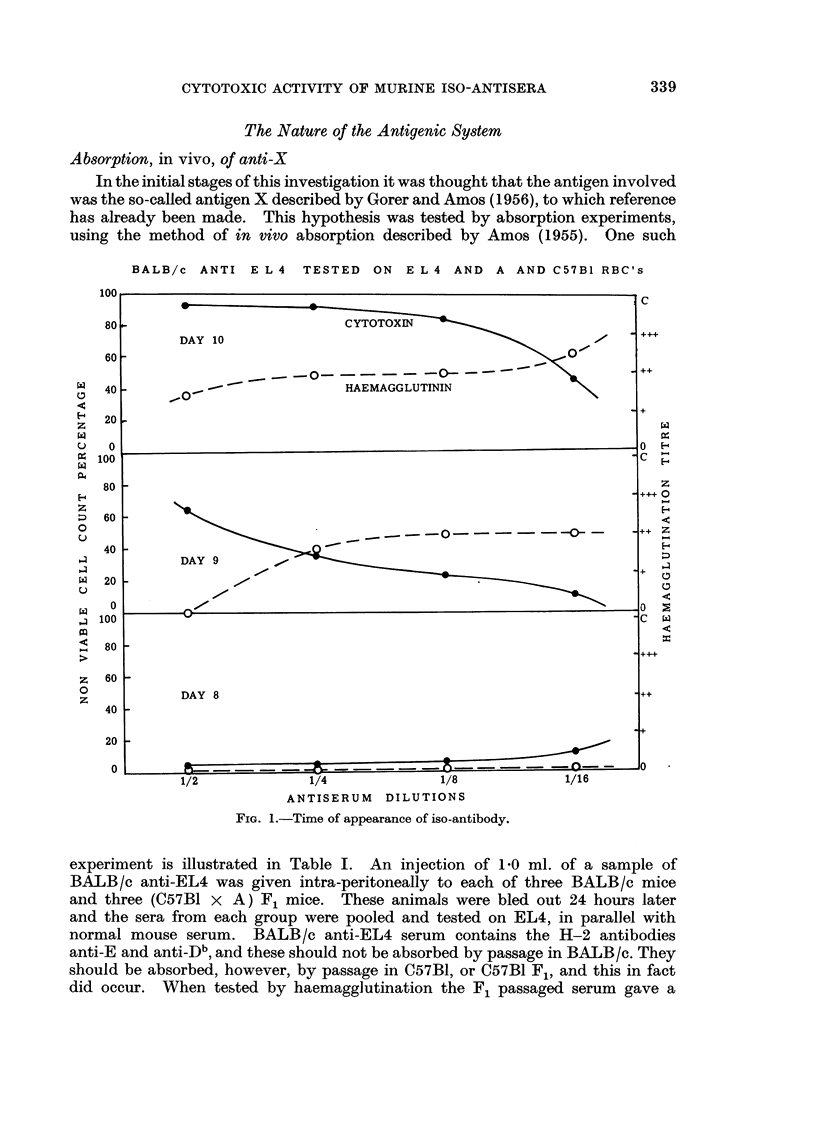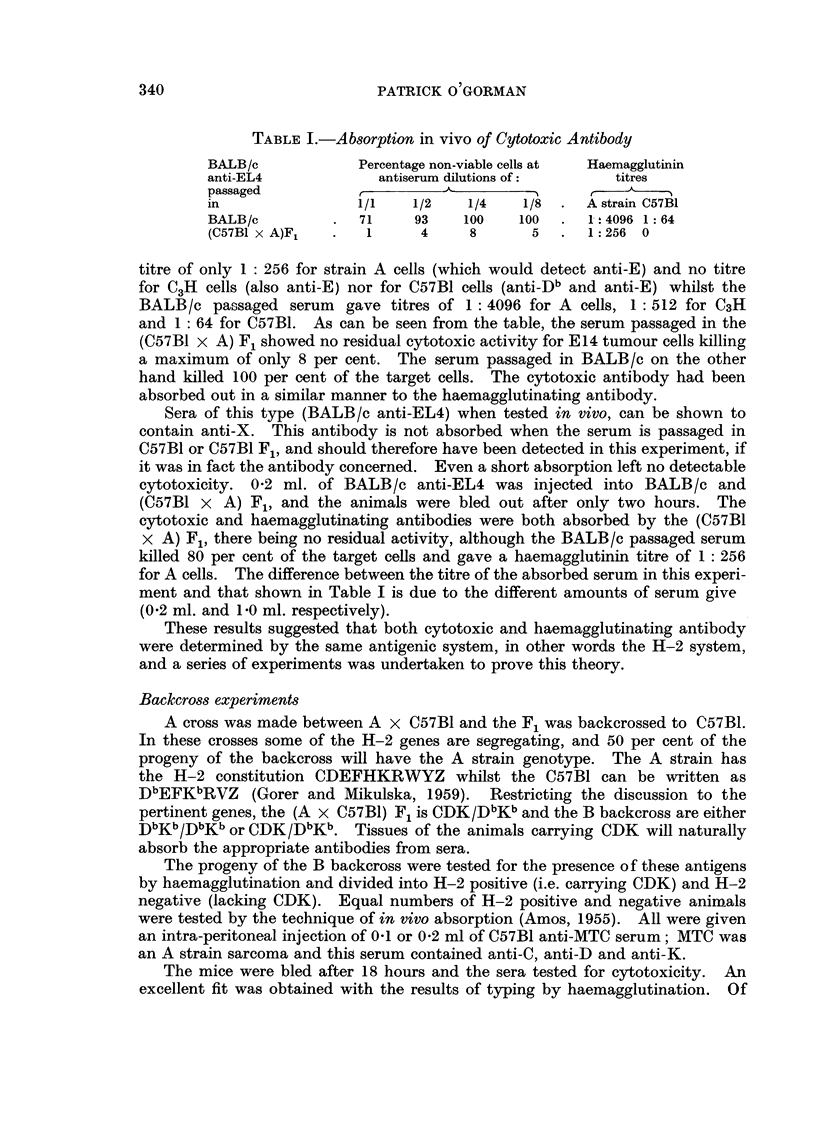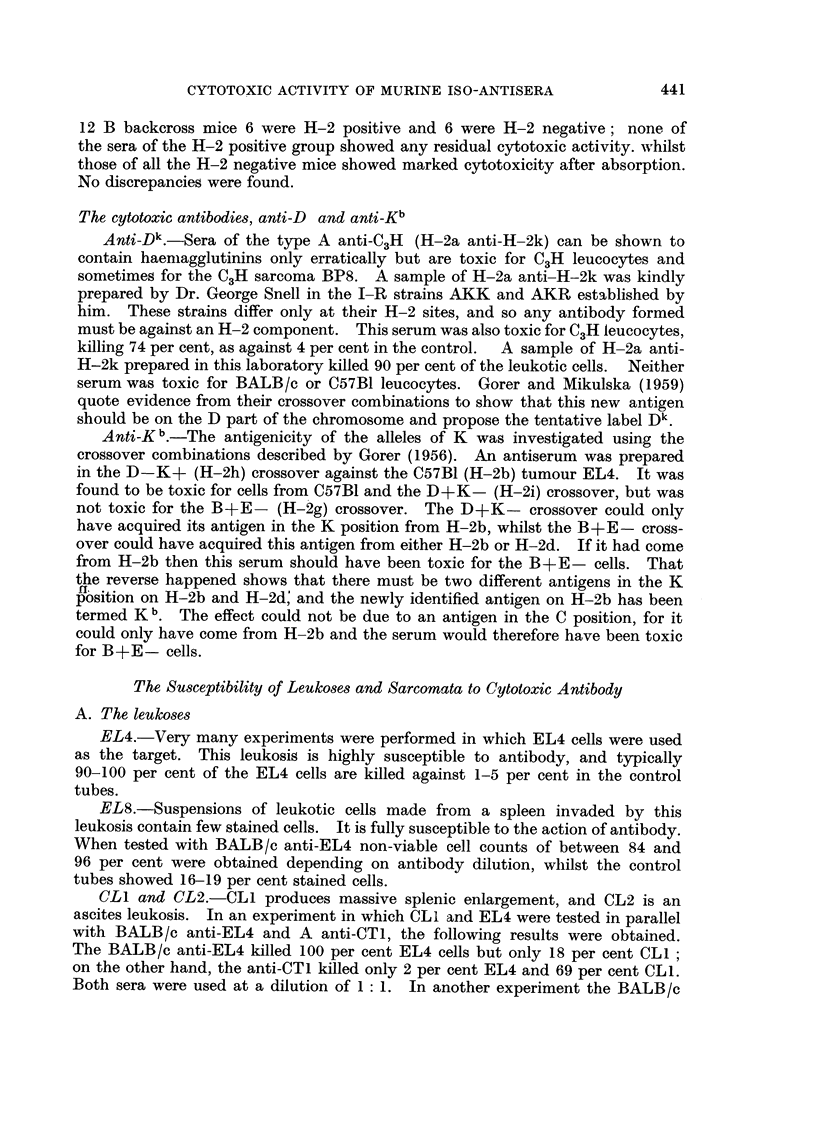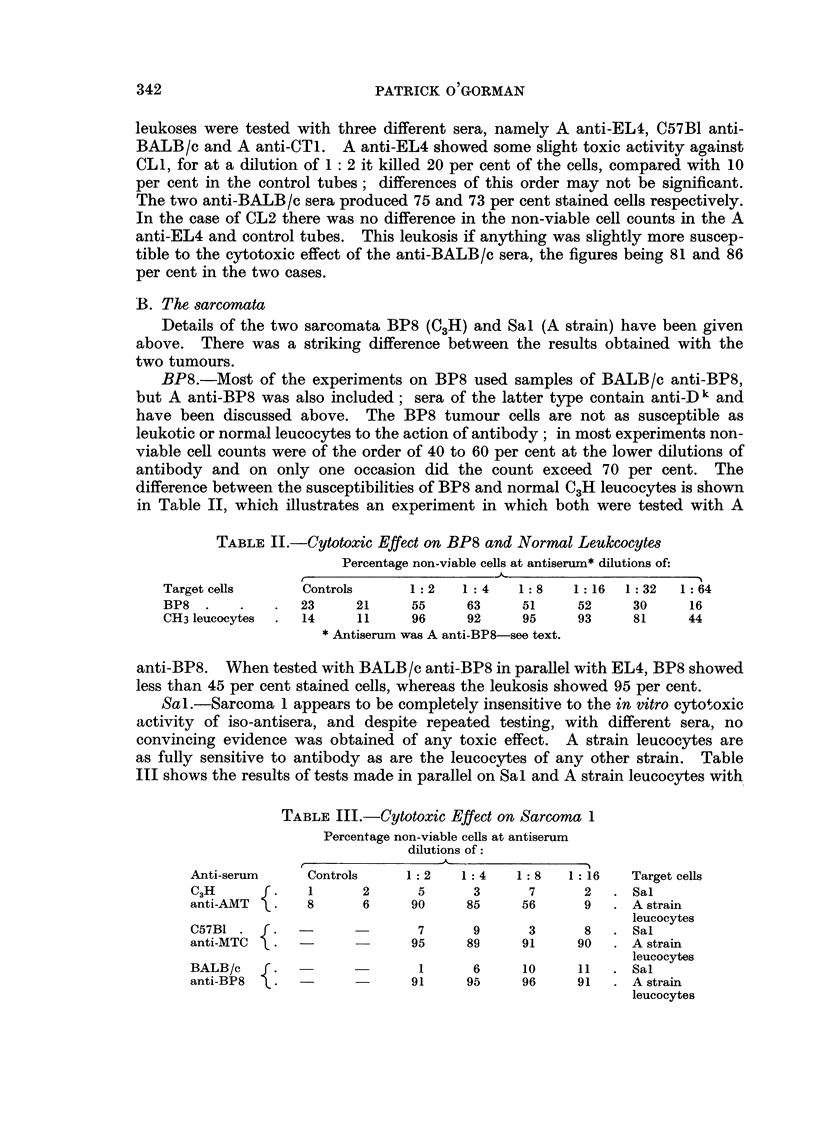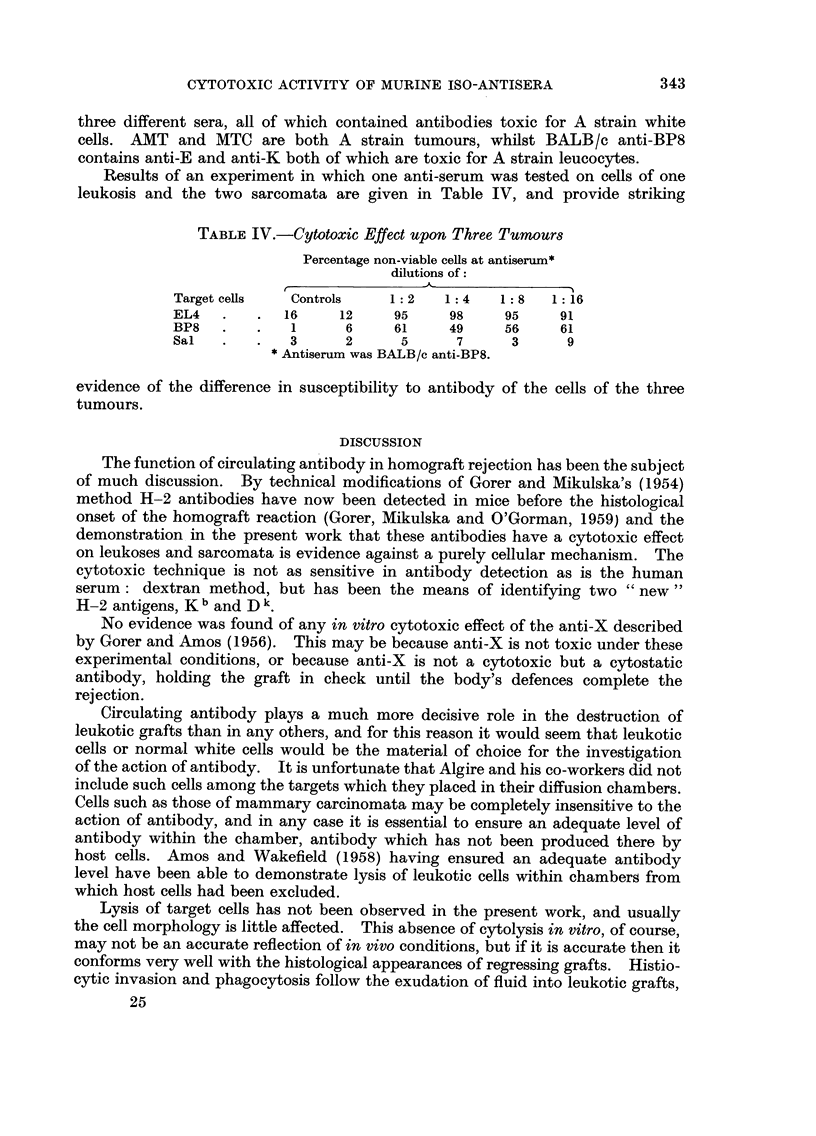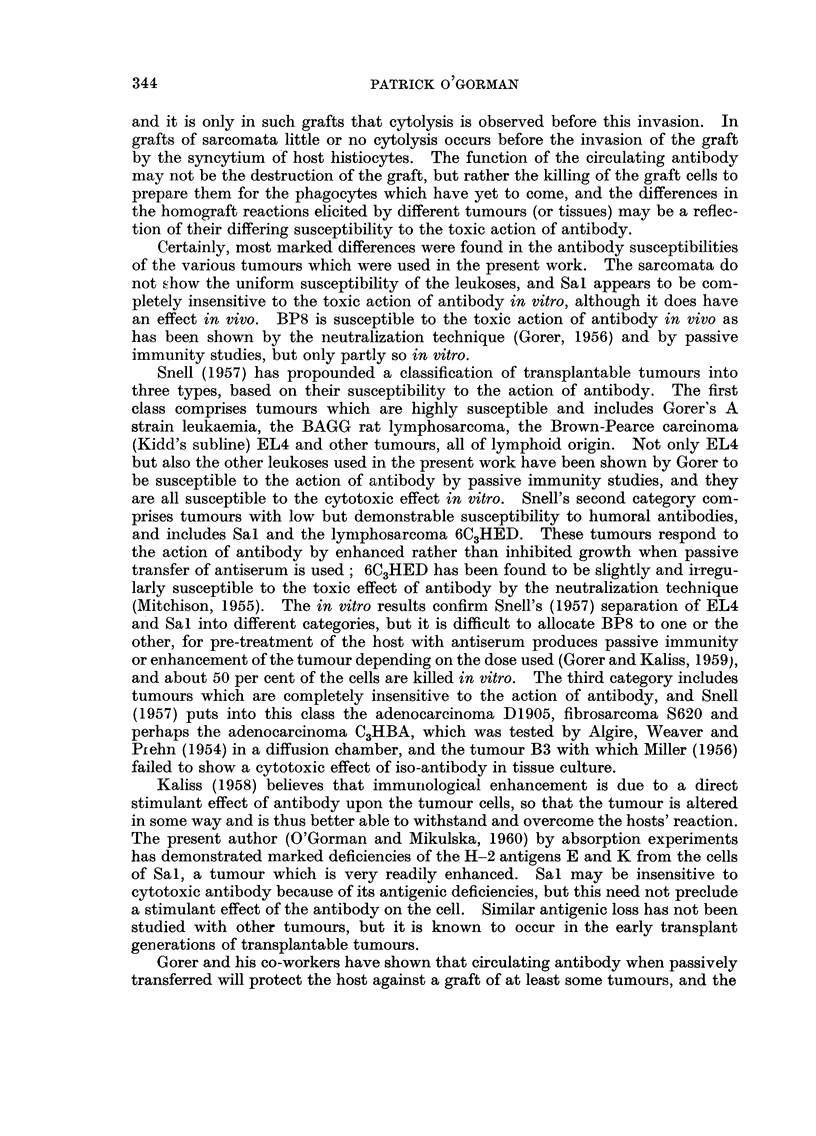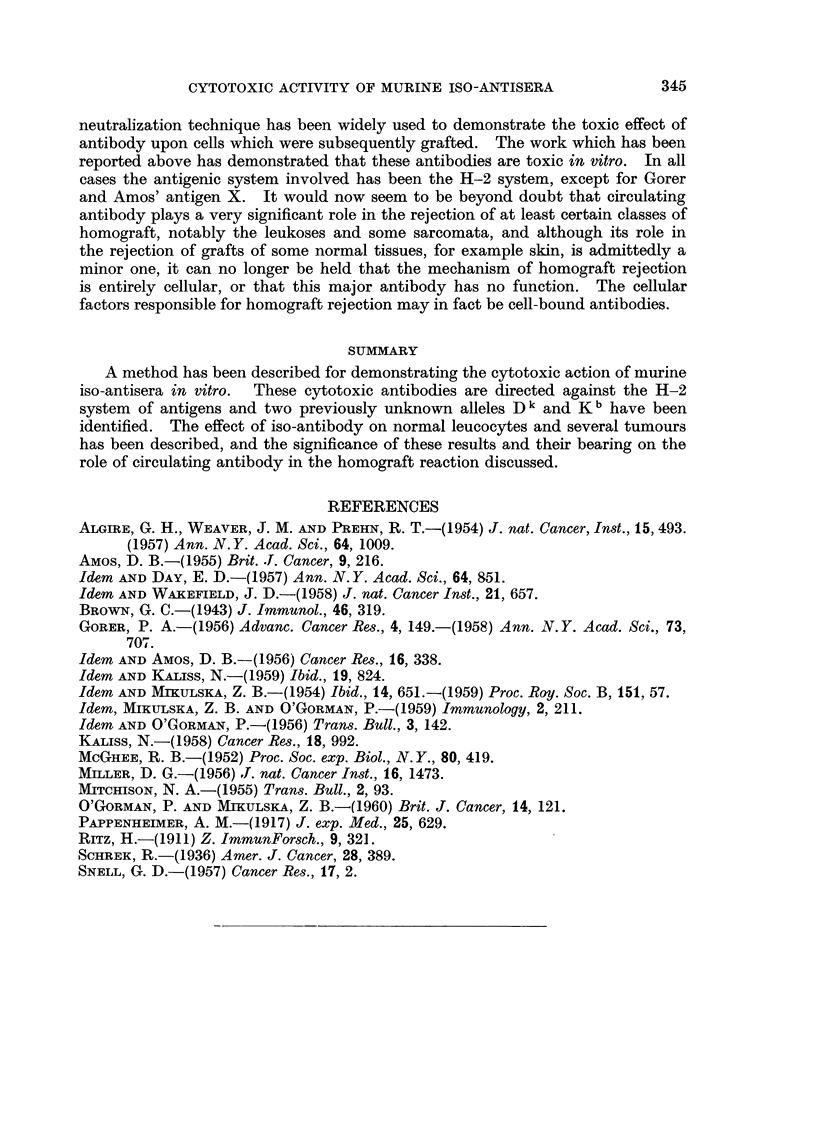# The In Vitro Cytotoxic Activity of Murine Iso-antisera on Normal and Malignant Cells*

**DOI:** 10.1038/bjc.1960.40

**Published:** 1960-06

**Authors:** Patrick O'Gorman


### THE *IN VITRO* CYTOTOXIC ACTIVITY OF MURINE ISO-ANTISERA ON NORMAL AND MALIGNANT CELLS*

PATRICK O'GORMAN†

*From the Department of Experimental Pathology, Guy's Hospital Medical School*

Received for publication April 13, 1960

IN mice passive immunity to leukaemic homografts has been obtained by transfer of sera containing iso-antibodies against both the H–2 antigens and the so-called X-type antigens, examples of which have been found in several leukoses (Amos and Day, 1957 ; Gorer and Amos, 1956 ; Gorer, personal communication). Histologically, leukaemic grafts can be seen to be destroyed by a massive exudation of fluid into the graft on the fourth and fifth day ; thereafter host cells appear which act only as scavengers (Gorer, 1958). Sarcomatous grafts, on the other hand, are invaded by a syncytium of host cells which engulf and destroy the graft. The antigens of the H–2 system are the major antigenic factors controlling the fate of homografts in mice, and are determined by a long series of alleles on the ninth chromosome. Present knowledge of this system has been reviewed by Gorer and Mikulska (1959).

These results suggested that iso-antibodies play an important part in homograft rejection, particularly of leukotic grafts, and might have a detectable effect *in vitro*. There have been few purely *in vitro* studies of this effect. Miller (1956) failed to show any effect upon cells growing in tissue culture of exposure to iso-antibody ; the cells were of epithelial origin and the presence of active complement was not ensured. Mouse serum is usually devoid of complementary activity *in vitro*, and Ritz (1911) stated that mouse serum was deficient in the " end-piece " of complement, whilst Brown (1943) believed it to lack the second component, C′2. McGhee (1952) demonstrated murine complement *in vitro* and convincing evidence of its activity *in vivo* has been provided by the experiments with diffusion chambers reported by Algire, Weaver and Prehn (1957), who obtained lysis of Hela cells and by Amos and Wakefield (1958) who demonstrated lysis of mouse leukotic cells.

Schrek (1936) has described a method of distinguishing viable and non-viable cells in a suspension by means of differential staining, using eosin, methylene blue and trypan blue, dyes which stain non-viable cells, whilst viable cells are not affected, as Pappenheimer (1917) had shown many years before. A technique for studying the effect of murine iso-antisera *in vitro* was briefly reported by Gorer and the present author (Gorer and O'Gorman, 1956) which used Schrek's method. The technique did not prove completely reliable in further use and a more satisfactory modification is now presented, together with a detailed study of the nature of the antibody, of the antigenic system involved and of the effect of

* This work has formed part of a thesis accepted by the University of London for the degree of Doctor of Medicine.

† Present address : Group Laboratory, Lewisham Hospital, London, S.E.13.

cytotoxic iso-antibody upon normal leucocytes and upon several leukoses and two sarcomata.

### MATERIALS

*Mice.*—The mice used were from the inbred lines Strain A, BALB/c, $C_3H$ and C57Bl, maintained in the laboratory of Dr. P. A. Gorer, and also the progeny of the backcross (A $\times$ C57Bl) F, $\times$ C57Bl.

*Complement.*—Guinea-pig complement was used, obtained by cardiac puncture and stored at $-20°$ C. until needed.

*Tumours.*—Two ascites leukoses were used, EL4 (C57Bl strain) and CL2 (BALB/c). EL4 has been maintained by transplantation for many years, and will not grow subcutaneously in foreign strains, but sometimes will do so intraperitoneally. CL2 is of recent origin and is strain specific. Two other leukoses CL1 (BALB/c) and EL8 (C57Bl) are of recent origin and are strain specific, but produce generalized leukoses with massive enlargement of the spleen. Three ascites sarcomata weie used, BP8 ($C_3H$ strain) and Sa1 and MTC 1A (both A strain). All of these tumours sometimes grow progressively if inoculated intraperitoneally in large doses into foreign strains. In moderate dosage, subcutaneously, they are strain specific. Three other strain specific tumours were used—the mammary carcinomata EMT (C57Bl), AMT (A strain) and CT1 (BALB/c) and the leukosis EL5 ; all were maintained as solid subcutaneous growths.

*Iso-antisera.*—Details of the hyper-immune iso-antisera used will be given at the appropriate places in the text.

*Dyes.*—Both eosin and trypan blue were used at a concentration of 1/2000 in Tyrode's solution, filtered after preparation. No significant difference was observed in the results of parallel experiments using the two dyes, but trypan blue gives more clear-cut staining.

### METHODS

### *Preparation of cell suspensions*

1. *From ascites tumours.*—The ascites was diluted in Ringer's solution in the case of the non-haemorrhagic leukotic ascites, and in 3 per cent sodium citrate in the case of the haemorrhagic sarcomatous ascites. Total cell counts were made at once, and the dilution adjusted with normal mouse serum and Ringer's solution to give a final count of approximately 30,000/mm.$^3$ in 1 : 5 normal mouse serum. Viability counts gave from 0–5 per cent stained cells in the case of the ascites leukoses, and about 0–10 per cent in the ascites sarcomata.

2. *From spleen.*—The same method was used for both normal and leukotic spleens. The animal was killed with chloroform, the abdomen opened, the spleen exposed and the splenic artery and vein were cut with fine scissors. A 10 ml. syringe was fitted with a fine intra-dermal (No. 26 G.) needle and filled with sterile Ringer's solution. The point of the needle was introduced under the capsule of the spleen and light pressure applied to the plunger. Immediately a small white area appeared around the needle point, which extended as the pressure was maintained. After about 1 ml. had been injected the needle was withdrawn and reinserted in a different position. In this way the whole spleen was gradually perfused. If the splenic vessels are not divided, the liver and kidneys can be perfused at the same time, and this is probably a more convenient way of perfusing these organs than by cannulation of the appropriate artery or by intra-cardiac injection. After perfusion the spleen was excised, freed from fat and finely minced with small scissors and suspended in 1 : 5 normal mouse serum. Small clumps were broken up by gentle pipetting, and the larger clumps which remained were allowed to settle out. The concentration was adjusted to a final figure of approximately 30,000 leucocytes/$mm.^3$ Viability counts usually showed about 5–10 per cent stained cells.

*Titration of complement*

Undiluted guinea-pig serum was often toxic to the target cells whilst its complementary activity declined rapidly on dilution. It was therefore titrated in terms of its ability to complement the cytotoxic activity of iso-antisera. A serum known to be toxic for the target cells was used at a dilution of 1 : 5 and the complement was titrated in the dilution range 1 : 1 to 1 : 4 ; it was seldom active at greater dilutions. Controls of normal serum and another iso-antiserum known not to be toxic for the target cells were used.

*Antibody titration*

The cytotoxic activity of an antiserum was usually investigated in the form of a titration, to exclude zoning.

Doubling dilutions of each antiserum to be tested were made in 1 : 5 normal mouse serum. Control tubes, containing only the diluted normal serum, were included. A volume (0·05 ml.) of cell suspension was added to each tube and finally a volume of diluted complement. The rack was incubated at 37° C. for 45–75 minutes, except in the experiments on the timing of the reaction. After incubation dye was immediately added to all the tubes to make a final dilution of 1 : 10. The tubes were left for a few minutes before counting to ensure correct staining.

Two methods of reading the titration were employed. In the first, differential counts were made on each tube. In the second, the two control tubes were counted and the cells from the rest of the row were streaked across a glass slide and inspected. The results were expressed as +++, ++ etc., the term AC being used when the effect was almost complete and there were virtually no viable cells to be seen; a negative was scored when the number of stained cells approached that in the control tubes. This method is entirely satisfactory for the routine titration of a serum, but the method expressing the result as a percentage viable count was found to be more delicate when comparative work was being done. Results obtained by the two methods are strictly comparable.

*Haemagglutination*

The " human serum : dextran " technique of haemagglutination described by Gorer and Milulska (1954) was used for genetic studies and titration of sera.

In vivo *absorption*

Amos (1955) has described the *in vivo* absorption of antibody by mice possessing the appropriate antigen. If a potent serum such as BALB/c anti-C57Bl, is injected intra-peritoneally into C57Bl mice, the haemagglutinin titre in the recipients' serum falls rapidly and will not be detectable after two hours, even if as much as 0·5 ml. of antiserum is injected. The absorption in all cases is specific and non-specific absorption does not occur.

### RESULTS

*The morphology of the damaged cells*

There was considerable variation in the appearance of the stained cells. Whilst some retained a normal rounded outline, in which the nucleus could barely be seen, other cells became swollen, sometimes grossly so. The outline of these cells became irregular and indistinct and occasionally the cell membrane appeared to have developed bubbles. In the more severely affected cells the nucleus could be clearly seen when trypan blue was used as the stain. Actual lysis of the cells was not seen.

*Effect on normal and malignant cells*

No significant difference could be detected between the results obtained using normal or leukotic white cells, but ascites leukoses were preferable as the suspensions contained less debris and fewer stained cells than those made from spleen or sarcomatous ascites.

*The effect of heat*

Heating the antiserum at 56° C. for 30 minutes was without effect on the titre.

*Rate of reaction*

Murine iso-antisera appear to be toxic to homologous cells even after very short exposure, in the presence of active complement. In an experiment in which BALB/c anti-BP8 was tested on normal $C_3H$ leucocytes, 90 per cent of the target cells were stained after only 10 minutes' incubation at 37° C. The control at this stage contained only 5 per cent non-viable cells. After 20 minutes' incubation 95 per cent of the cells were dead ; the increase is probably not significant. On the other hand, prolonged incubation, i.e. for more than $1\frac{1}{2}$ hours produced high numbers of dead cells in the control tubes.

*Time of appearance of cytotoxic antibody*

Groups of four mice were bled on successive days after inoculation with tumour suspension. The blood from the individuals of each group was pooled and the cytotoxic activity of the serum tested on appropriate cells. The haemagglutinating titre was determined at the same time.

The results of one such experiment are expressed graphically in Fig. 1. The animals were BALB/c, inoculated subcutaneously with EL4. The sera were tested for cytotoxicity on EL4, and for haemagglutinating activity on A and C57Bl red cells. As can be seen from the graphs neither cytotoxin nor haemagglutinin was present on the eighth day after inoculation. Both appeared on the ninth day, and the titres rose together on the same day.

### *The Nature of the Antigenic System*

*Absorption,* in vivo, *of anti-X*

In the initial stages of this investigation it was thought that the antigen involved was the so-called antigen X described by Gorer and Amos (1956), to which reference has already been made. This hypothesis was tested by absorption experiments, using the method of *in vivo* absorption described by Amos (1955). One such

FIG. 1.—Time of appearance of iso-antibody.

experiment is illustrated in Table I. An injection of 1·0 ml. of a sample of BALB/c anti-EL4 was given intra-peritoneally to each of three BALB/c mice and three (C57Bl × A) $F_1$ mice. These animals were bled out 24 hours later and the sera from each group were pooled and tested on EL4, in parallel with normal mouse serum. BALB/c anti-EL4 serum contains the H–2 antibodies anti-E and anti-$D^b$, and these should not be absorbed by passage in BALB/c. They should be absorbed, however, by passage in C57Bl, or C57Bl $F_1$, and this in fact did occur. When tested by haemagglutination the $F_1$ passaged serum gave a

TABLE I.—*Absorption* in vivo *of Cytotoxic Antibody*

| BALB/c anti-EL4 passaged in | Percentage non-viable cells at antiserum dilutions of: | | | | Haemagglutinin titres | |
|---|---|---|---|---|---|---|
| | 1/1 | 1/2 | 1/4 | 1/8 | A strain | C57Bl |
| BALB/c | 71 | 93 | 100 | 100 | 1 : 4096 | 1 : 64 |
| (C57Bl × A)$F_1$ | 1 | 4 | 8 | 5 | 1 : 256 | 0 |

titre of only 1 : 256 for strain A cells (which would detect anti-E) and no titre for $C_3H$ cells (also anti-E) nor for C57Bl cells (anti-$D^b$ and anti-E) whilst the BALB/c passaged serum gave titres of 1 : 4096 for A cells, 1 : 512 for C3H and 1 : 64 for C57Bl. As can be seen from the table, the serum passaged in the (C57Bl × A) $F_1$ showed no residual cytotoxic activity for E14 tumour cells killing a maximum of only 8 per cent. The serum passaged in BALB/c on the other hand killed 100 per cent of the target cells. The cytotoxic antibody had been absorbed out in a similar manner to the haemagglutinating antibody.

Sera of this type (BALB/c anti-EL4) when tested *in vivo*, can be shown to contain anti-X. This antibody is not absorbed when the serum is passaged in C57Bl or C57Bl $F_1$, and should therefore have been detected in this experiment, if it was in fact the antibody concerned. Even a short absorption left no detectable cytotoxicity. 0·2 ml. of BALB/c anti-EL4 was injected into BALB/c and (C57Bl × A) $F_1$, and the animals were bled out after only two hours. The cytotoxic and haemagglutinating antibodies were both absorbed by the (C57Bl × A) $F_1$, there being no residual activity, although the BALB/c passaged serum killed 80 per cent of the target cells and gave a haemagglutinin titre of 1 : 256 for A cells. The difference between the titre of the absorbed serum in this experiment and that shown in Table I is due to the different amounts of serum give (0·2 ml. and 1·0 ml. respectively).

These results suggested that both cytotoxic and haemagglutinating antibody were determined by the same antigenic system, in other words the H–2 system, and a series of experiments was undertaken to prove this theory.

*Backcross experiments*

A cross was made between A × C57Bl and the $F_1$ was backcrossed to C57Bl. In these crosses some of the H–2 genes are segregating, and 50 per cent of the progeny of the backcross will have the A strain genotype. The A strain has the H–2 constitution CDEFHKRWYZ whilst the C57Bl can be written as $D^bEFK^bRVZ$ (Gorer and Mikulska, 1959). Restricting the discussion to the pertinent genes, the (A × C57Bl) $F_1$ is $CDK/D^bK^b$ and the B backcross are either $D^bK^b/D^bK^b$ or $CDK/D^bK^b$. Tissues of the animals carrying CDK will naturally absorb the appropriate antibodies from sera.

The progeny of the B backcross were tested for the presence of these antigens by haemagglutination and divided into H–2 positive (i.e. carrying CDK) and H–2 negative (lacking CDK). Equal numbers of H–2 positive and negative animals were tested by the technique of *in vivo* absorption (Amos, 1955). All were given an intra-peritoneal injection of 0·1 or 0·2 ml of C57Bl anti-MTC serum ; MTC was an A strain sarcoma and this serum contained anti-C, anti-D and anti-K.

The mice were bled after 18 hours and the sera tested for cytotoxicity. An excellent fit was obtained with the results of typing by haemagglutination. Of

12 B backcross mice 6 were H–2 positive and 6 were H–2 negative; none of the sera of the H–2 positive group showed any residual cytotoxic activity. whilst those of all the H–2 negative mice showed marked cytotoxicity after absorption. No discrepancies were found.

*The cytotoxic antibodies, anti-D and anti-$K^b$*

*Anti-$D^k$*.—Sera of the type A anti-$C_3H$ (H–2a anti-H–2k) can be shown to contain haemagglutinins only erratically but are toxic for $C_3H$ leucocytes and sometimes for the $C_3H$ sarcoma BP8. A sample of H–2a anti–H–2k was kindly prepared by Dr. George Snell in the I–R strains AKK and AKR established by him. These strains differ only at their H–2 sites, and so any antibody formed must be against an H–2 component. This serum was also toxic for $C_3H$ leucocytes, killing 74 per cent, as against 4 per cent in the control. A sample of H–2a anti-H–2k prepared in this laboratory killed 90 per cent of the leukotic cells. Neither serum was toxic for BALB/c or C57Bl leucocytes. Gorer and Mikulska (1959) quote evidence from their crossover combinations to show that this new antigen should be on the D part of the chromosome and propose the tentative label $D^k$.

*Anti-$K^b$*.—The antigenicity of the alleles of K was investigated using the crossover combinations described by Gorer (1956). An antiserum was prepared in the D—K+ (H–2h) crossover against the C57Bl (H–2b) tumour EL4. It was found to be toxic for cells from C57Bl and the D+K— (H–2i) crossover, but was not toxic for the B+E— (H–2g) crossover. The D+K— crossover could only have acquired its antigen in the K position from H–2b, whilst the B+E— crossover could have acquired this antigen from either H–2b or H–2d. If it had come from H–2b then this serum should have been toxic for the B+E— cells. That the reverse happened shows that there must be two different antigens in the K position on H–2b and H–2d, and the newly identified antigen on H–2b has been termed $K^b$. The effect could not be due to an antigen in the C position, for it could only have come from H–2b and the serum would therefore have been toxic for B+E— cells.

*The Susceptibility of Leukoses and Sarcomata to Cytotoxic Antibody*

A. *The leukoses*

*EL4*.—Very many experiments were performed in which EL4 cells were used as the target. This leukosis is highly susceptible to antibody, and typically 90–100 per cent of the EL4 cells are killed against 1–5 per cent in the control tubes.

*EL8*.—Suspensions of leukotic cells made from a spleen invaded by this leukosis contain few stained cells. It is fully susceptible to the action of antibody. When tested with BALB/c anti-EL4 non-viable cell counts of between 84 and 96 per cent were obtained depending on antibody dilution, whilst the control tubes showed 16–19 per cent stained cells.

*CL1 and CL2*.—CL1 produces massive splenic enlargement, and CL2 is an ascites leukosis. In an experiment in which CL1 and EL4 were tested in parallel with BALB/c anti-EL4 and A anti-CT1, the following results were obtained. The BALB/c anti-EL4 killed 100 per cent EL4 cells but only 18 per cent CL1; on the other hand, the anti-CT1 killed only 2 per cent EL4 and 69 per cent CL1. Both sera were used at a dilution of 1 : 1. In another experiment the BALB/c leukoses were tested with three different sera, namely A anti-EL4, C57Bl anti-BALB/c and A anti-CT1. A anti-EL4 showed some slight toxic activity against CL1, for at a dilution of 1 : 2 it killed 20 per cent of the cells, compared with 10 per cent in the control tubes; differences of this order may not be significant. The two anti-BALB/c sera produced 75 and 73 per cent stained cells respectively. In the case of CL2 there was no difference in the non-viable cell counts in the A anti-EL4 and control tubes. This leukosis if anything was slightly more susceptible to the cytotoxic effect of the anti-BALB/c sera, the figures being 81 and 86 per cent in the two cases.

B. *The sarcomata*

Details of the two sarcomata BP8 ($C_3H$) and Sa1 (A strain) have been given above. There was a striking difference between the results obtained with the two tumours.

*BP*8.—Most of the experiments on BP8 used samples of BALB/c anti-BP8, but A anti-BP8 was also included; sera of the latter type contain anti-$D^k$ and have been discussed above. The BP8 tumour cells are not as susceptible as leukotic or normal leucocytes to the action of antibody; in most experiments non-viable cell counts were of the order of 40 to 60 per cent at the lower dilutions of antibody and on only one occasion did the count exceed 70 per cent. The difference between the susceptibilities of BP8 and normal $C_3H$ leucocytes is shown in Table II, which illustrates an experiment in which both were tested with A anti-BP8. When tested with BALB/c anti-BP8 in parallel with EL4, BP8 showed less than 45 per cent stained cells, whereas the leukosis showed 95 per cent.

TABLE II.—*Cytotoxic Effect on BP*8 *and Normal Leukcocytes*

| Target cells | Percentage non-viable cells at antiserum* dilutions of: Controls | | 1 : 2 | 1 : 4 | 1 : 8 | 1 : 16 | 1 : 32 | 1 : 64 |
|---|---|---|---|---|---|---|---|---|
| BP8 | 23 | 21 | 55 | 63 | 51 | 52 | 30 | 16 |
| CH3 leucocytes | 14 | 11 | 96 | 92 | 95 | 93 | 81 | 44 |

* Antiserum was A anti-BP8—see text.

*Sa*1.—Sarcoma 1 appears to be completely insensitive to the *in vitro* cytotoxic activity of iso-antisera, and despite repeated testing, with different sera, no convincing evidence was obtained of any toxic effect. A strain leucocytes are as fully sensitive to antibody as are the leucocytes of any other strain. Table III shows the results of tests made in parallel on Sa1 and A strain leucocytes with

TABLE III.—*Cytotoxic Effect on Sarcoma* 1

| Anti-serum | Percentage non-viable cells at antiserum dilutions of: Controls | | 1 : 2 | 1 : 4 | 1 : 8 | 1 : 16 | Target cells |
|---|---|---|---|---|---|---|---|
| $C_3H$ anti-AMT | 1 | 2 | 5 | 3 | 7 | 2 | Sa1 |
| | 8 | 6 | 90 | 85 | 56 | 9 | A strain leucocytes |
| C57Bl anti-MTC | — | — | 7 | 9 | 3 | 8 | Sa1 |
| | — | — | 95 | 89 | 91 | 90 | A strain leucocytes |
| BALB/c anti-BP8 | — | — | 1 | 6 | 10 | 11 | Sa1 |
| | — | — | 91 | 95 | 96 | 91 | A strain leucocytes |

three different sera, all of which contained antibodies toxic for A strain white cells. AMT and MTC are both A strain tumours, whilst BALB/c anti-BP8 contains anti-E and anti-K both of which are toxic for A strain leucocytes.

Results of an experiment in which one anti-serum was tested on cells of one leukosis and the two sarcomata are given in Table IV, and provide striking

TABLE IV.—*Cytotoxic Effect upon Three Tumours*

| Target cells | Percentage non-viable cells at antiserum* dilutions of: | | | | | |
|---|---|---|---|---|---|---|
| | Controls | | 1 : 2 | 1 : 4 | 1 : 8 | 1 : 16 |
| EL4 | 16 | 12 | 95 | 98 | 95 | 91 |
| BP8 | 1 | 6 | 61 | 49 | 56 | 61 |
| Sal | 3 | 2 | 5 | 7 | 3 | 9 |

* Antiserum was BALB/c anti-BP8.

evidence of the difference in susceptibility to antibody of the cells of the three tumours.

### DISCUSSION

The function of circulating antibody in homograft rejection has been the subject of much discussion. By technical modifications of Gorer and Mikulska's (1954) method H–2 antibodies have now been detected in mice before the histological onset of the homograft reaction (Gorer, Mikulska and O'Gorman, 1959) and the demonstration in the present work that these antibodies have a cytotoxic effect on leukoses and sarcomata is evidence against a purely cellular mechanism. The cytotoxic technique is not as sensitive in antibody detection as is the human serum : dextran method, but has been the means of identifying two " new " H–2 antigens, $K^b$ and $D^k$.

No evidence was found of any *in vitro* cytotoxic effect of the anti-X described by Gorer and Amos (1956). This may be because anti-X is not toxic under these experimental conditions, or because anti-X is not a cytotoxic but a cytostatic antibody, holding the graft in check until the body's defences complete the rejection.

Circulating antibody plays a much more decisive role in the destruction of leukotic grafts than in any others, and for this reason it would seem that leukotic cells or normal white cells would be the material of choice for the investigation of the action of antibody. It is unfortunate that Algire and his co-workers did not include such cells among the targets which they placed in their diffusion chambers. Cells such as those of mammary carcinomata may be completely insensitive to the action of antibody, and in any case it is essential to ensure an adequate level of antibody within the chamber, antibody which has not been produced there by host cells. Amos and Wakefield (1958) having ensured an adequate antibody level have been able to demonstrate lysis of leukotic cells within chambers from which host cells had been excluded.

Lysis of target cells has not been observed in the present work, and usually the cell morphology is little affected. This absence of cytolysis *in vitro*, of course, may not be an accurate reflection of *in vivo* conditions, but if it is accurate then it conforms very well with the histological appearances of regressing grafts. Histiocytic invasion and phagocytosis follow the exudation of fluid into leukotic grafts, and it is only in such grafts that cytolysis is observed before this invasion. In grafts of sarcomata little or no cytolysis occurs before the invasion of the graft by the syncytium of host histiocytes. The function of the circulating antibody may not be the destruction of the graft, but rather the killing of the graft cells to prepare them for the phagocytes which have yet to come, and the differences in the homograft reactions elicited by different tumours (or tissues) may be a reflection of their differing susceptibility to the toxic action of antibody.

Certainly, most marked differences were found in the antibody susceptibilities of the various tumours which were used in the present work. The sarcomata do not show the uniform susceptibility of the leukoses, and Sa1 appears to be completely insensitive to the toxic action of antibody *in vitro*, although it does have an effect *in vivo*. BP8 is susceptible to the toxic action of antibody *in vivo* as has been shown by the neutralization technique (Gorer, 1956) and by passive immunity studies, but only partly so *in vitro*.

Snell (1957) has propounded a classification of transplantable tumours into three types, based on their susceptibility to the action of antibody. The first class comprises tumours which are highly susceptible and includes Gorer's A strain leukaemia, the BAGG rat lymphosarcoma, the Brown-Pearce carcinoma (Kidd's subline) EL4 and other tumours, all of lymphoid origin. Not only EL4 but also the other leukoses used in the present work have been shown by Gorer to be susceptible to the action of antibody by passive immunity studies, and they are all susceptible to the cytotoxic effect *in vitro*. Snell's second category comprises tumours with low but demonstrable susceptibility to humoral antibodies, and includes Sa1 and the lymphosarcoma $6C_3HED$. These tumours respond to the action of antibody by enhanced rather than inhibited growth when passive transfer of antiserum is used ; $6C_3HED$ has been found to be slightly and irregularly susceptible to the toxic effect of antibody by the neutralization technique (Mitchison, 1955). The *in vitro* results confirm Snell's (1957) separation of EL4 and Sa1 into different categories, but it is difficult to allocate BP8 to one or the other, for pre-treatment of the host with antiserum produces passive immunity or enhancement of the tumour depending on the dose used (Gorer and Kaliss, 1959), and about 50 per cent of the cells are killed *in vitro*. The third category includes tumours which are completely insensitive to the action of antibody, and Snell (1957) puts into this class the adenocarcinoma D1905, fibrosarcoma S620 and perhaps the adenocarcinoma $C_3HBA$, which was tested by Algire, Weaver and Prehn (1954) in a diffusion chamber, and the tumour B3 with which Miller (1956) failed to show a cytotoxic effect of iso-antibody in tissue culture.

Kaliss (1958) believes that immunological enhancement is due to a direct stimulant effect of antibody upon the tumour cells, so that the tumour is altered in some way and is thus better able to withstand and overcome the hosts' reaction. The present author (O'Gorman and Mikulska, 1960) by absorption experiments has demonstrated marked deficiencies of the H–2 antigens E and K from the cells of Sa1, a tumour which is very readily enhanced. Sa1 may be insensitive to cytotoxic antibody because of its antigenic deficiencies, but this need not preclude a stimulant effect of the antibody on the cell. Similar antigenic loss has not been studied with other tumours, but it is known to occur in the early transplant generations of transplantable tumours.

Gorer and his co-workers have shown that circulating antibody when passively transferred will protect the host against a graft of at least some tumours, and the neutralization technique has been widely used to demonstrate the toxic effect of antibody upon cells which were subsequently grafted. The work which has been reported above has demonstrated that these antibodies are toxic *in vitro*. In all cases the antigenic system involved has been the H–2 system, except for Gorer and Amos' antigen X. It would now seem to be beyond doubt that circulating antibody plays a very significant role in the rejection of at least certain classes of homograft, notably the leukoses and some sarcomata, and although its role in the rejection of grafts of some normal tissues, for example skin, is admittedly a minor one, it can no longer be held that the mechanism of homograft rejection is entirely cellular, or that this major antibody has no function. The cellular factors responsible for homograft rejection may in fact be cell-bound antibodies.

### SUMMARY

A method has been described for demonstrating the cytotoxic action of murine iso-antisera *in vitro*. These cytotoxic antibodies are directed against the H–2 system of antigens and two previously unknown alleles $D^k$ and $K^b$ have been identified. The effect of iso-antibody on normal leucocytes and several tumours has been described, and the significance of these results and their bearing on the role of circulating antibody in the homograft reaction discussed.

### REFERENCES

ALGIRE, G. H., WEAVER, J. M. AND PREHN, R. T.—(1954) *J. nat. Cancer, Inst.*, **15**, 493. (1957) *Ann. N.Y. Acad. Sci.*, **64**, 1009.
AMOS, D. B.—(1955) *Brit. J. Cancer*, **9**, 216.
*Idem* AND DAY, E. D.—(1957) *Ann. N.Y. Acad. Sci.*, **64**, 851.
*Idem* AND WAKEFIELD, J. D.—(1958) *J. nat. Cancer Inst.*, **21**, 657.
BROWN, G. C.—(1943) *J. Immunol.*, **46**, 319.
GORER, P. A.—(1956) *Advanc. Cancer Res.*, **4**, 149.—(1958) *Ann. N.Y. Acad. Sci.*, **73**, 707.
*Idem* AND AMOS, D. B.—(1956) *Cancer Res.*, **16**, 338.
*Idem* AND KALISS, N.—(1959) *Ibid.*, **19**, 824.
*Idem* AND MIKULSKA, Z. B.—(1954) *Ibid.*, **14**, 651.—(1959) *Proc. Roy. Soc.* B, **151**, 57.
*Idem*, MIKULSKA, Z. B. AND O'GORMAN, P.—(1959) *Immunology*, **2**, 211.
*Idem* AND O'GORMAN, P.—(1956) *Trans. Bull.*, **3**, 142.
KALISS, N.—(1958) *Cancer Res.*, **18**, 992.
MCGHEE, R. B.—(1952) *Proc. Soc. exp. Biol., N.Y.*, **80**, 419.
MILLER, D. G.—(1956) *J. nat. Cancer Inst.*, **16**, 1473.
MITCHISON, N. A.—(1955) *Trans. Bull.*, **2**, 93.
O'GORMAN, P. AND MIKULSKA, Z. B.—(1960) *Brit. J. Cancer*, **14**, 121.
PAPPENHEIMER, A. M.—(1917) *J. exp. Med.*, **25**, 629.
RITZ, H.—(1911) *Z. ImmunForsch.*, **9**, 321.
SCHREK, R.—(1936) *Amer. J. Cancer*, **28**, 389.
SNELL, G. D.—(1957) *Cancer Res.*, **17**, 2.